# Supplementary material for: The C-terminal ZZ domain of the Drosophila ORB2 RNA-binding protein is required for spermatid individualization
Source: G3 (Bethesda). 2025 Oct 31;16(1):jkaf260. doi: 10.1093/g3journal/jkaf260 (PMC12774597; doi:10.1093/g3journal/jkaf260)
Supplement: jkaf260_Supplementary_Data [file jkaf260_supplementary_data.zip › Figure_S1_G3-2025-406341.pdf]

FIGURE S1

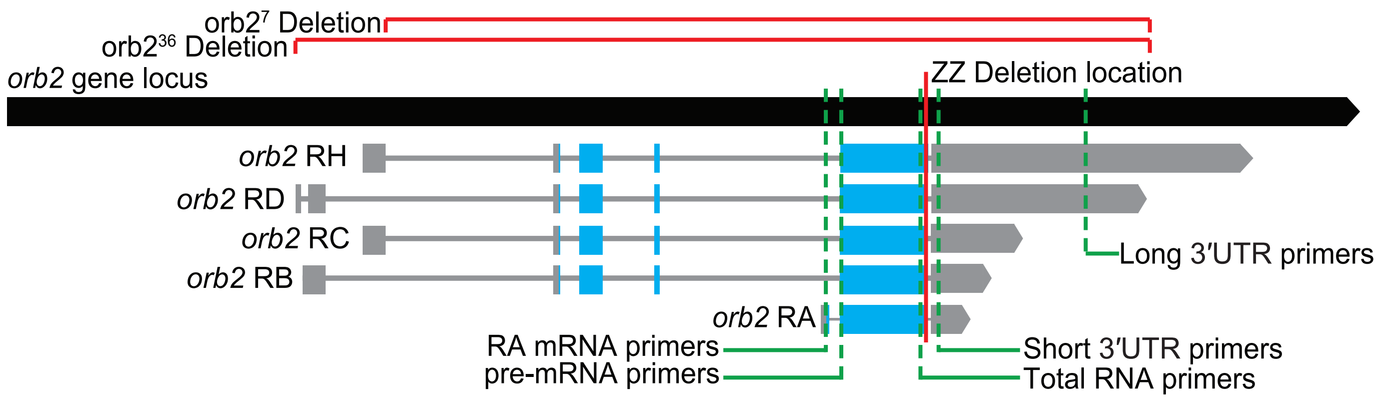

qPCR site	F primer (5′-3′)	R primer (5′-3′)	Amplicon Length
Pre-mRNA	agcctcttcatcttgttgtc	gctgttggtgctgatgga	159 bp[1]
Total RNA	cagatgtgcgacgagtgc	taacaccagcgaaaggggac	197 bp
Short 3′UTR	gggagtggcagcactagatg	ccccgggcaattgtcttttg	153 bp
Long 3′UTR	acagggcttaaagttcgcgt	ttgtttgcatgcaccgtagc	186 bp
RA mRNA	tgttaatttcatttgcggtggc[2]	gctgttggtgctgatgga	137 bp
ΔZZ	gccctatgtccttgtaacggcg[3]	ctcctcccgatcctgatgattct	141 bp

Note 1: pre-mRNA amplicon begins in an intron and crosses into the adjacent exon.
Note 2: RA mRNA F primer spans an exon-exon junction.
Note 3: ΔZZ F primer spans the ZZ deletion site.
