# Supplementary material for: The C-terminal ZZ domain of the Drosophila ORB2 RNA-binding protein is required for spermatid individualization
Source: G3 (Bethesda). 2025 Oct 31;16(1):jkaf260. doi: 10.1093/g3journal/jkaf260 (PMC12774597; doi:10.1093/g3journal/jkaf260)
Supplement: jkaf260_Supplementary_Data [file jkaf260_supplementary_data.zip › Figure_S1_Legend_G3-2025-406341.docx]

**Figure S1: Map of the *orb2* locus showing location and sequence of primers used in Figure 2a and b.** The *orb2* transcript isoforms B, C, D and H are as described in FlyBase (<https://flybase.org/reports/FBgn0264307#gene_model_products>).
